# Repair of critical diaphyseal defects of lower limbs by 3D printed porous Ti6Al4V scaffolds without additional bone grafting: a prospective clinical study

**DOI:** 10.1007/s10856-022-06685-0

**Published:** 2022-09-14

**Authors:** Bingchuan Liu, Guojin Hou, Zhongwei Yang, Xingcai Li, Yufeng Zheng, Peng Wen, Zhongjun Liu, Fang Zhou, Yun Tian

**Affiliations:** 1 Department of Orthopaedics, Peking University Third Hospital, Beijing, China; 2 Engineering Research Center of Bone and Joint Precision Medicine, Ministry of Education, Beijing, China; 3 School of Materials Science and Engineering, Peking University, Beijing, China; 4 Department of Mechanical Engineering, Tsinghua University, Beijing, China

## Abstract

The repair of critical diaphyseal defects of lower weight-bearing limbs is an intractable problem in clinical practice. From December 2017, we prospectively applied 3D printed porous Ti6Al4V scaffolds to reconstruct this kind of bone defect. All patients experienced a two-stage surgical process, including thorough debridement and scaffold implantation. With an average follow-up of 23.0 months, ten patients with 11 parts of bone defects were enrolled in this study. The case series included three females and seven males, their defect reasons included seven parts of osteomyelitis and four parts of aseptic nonunion. The bone defects located at femur (five parts) and tibia (six parts), with an average defect distance of 12.2 cm. Serial postoperative radiologic follow-ups displayed a continuous process of new bone growing and remodeling around the scaffold. One patient suffered tibial varus deformity, and he underwent a revision surgery. The other nine patients achieved scaffold stability. No scaffold breakage occurred. In conclusion, the implantation of 3D printed Ti6Al4V scaffold was feasible and effective to reconstruct critical bone defects of lower limbs without additional bone grafting.

**Figure:**
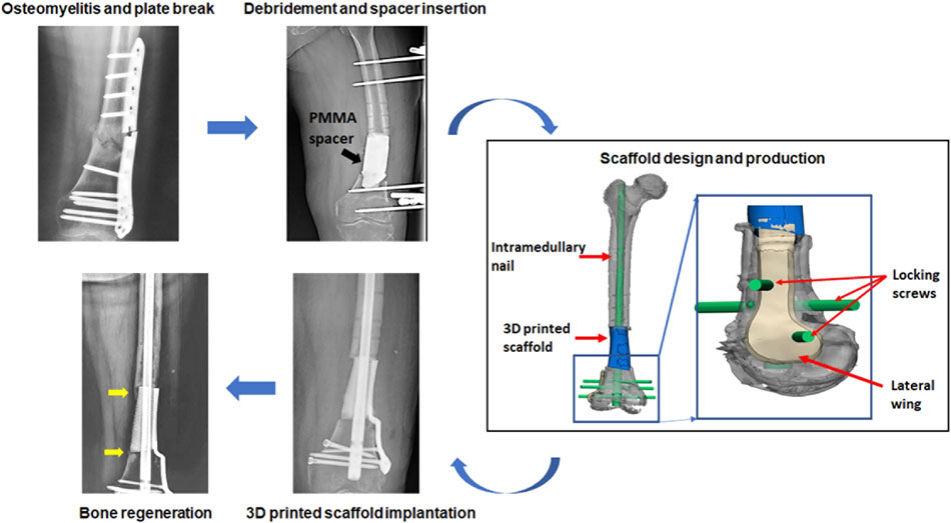
Graphical abstract

Graphical abstract

## Introduction

Critical bone defect is commonly defined as a large defect that may not heal spontaneously, which can be caused by severe fracture, infection, tumor resection, chronic nonunion, etc. [1–3]. Because of variable anatomical locations and geometries, conditions of periosteum and soft tissue, biomechanical environments, as well as patients’ basic conditions, its treatment still remains surgical, socioeconomic and investigative challenges [2–4]. In particular, diaphyseal bone defect of lower limbs appears to be more challengeable due to its higher requirement for weight-bearing and walking function. Although some approaches involving bone grafting, Ilizarov’s technology and Masquelet technique have revolutionized our ability to reconstruct bone defects [5–7], their application still possesses possibility of failure due to multiple reasons: (1) additional autogenous or allogeneic bone graft (ABG) implantation is inevitable in some conditions, which not only is irreverent to the original anatomical morphology, but also is associated with prolonged anesthesia duration, limited availability, and risk of infection and transmitting diseases [1, 5, 8]; (2) direct reconstruction of a critical defect with bone grafting may become hardly possible for early weight-bearing because its unstable biomechanical support, and gradual graft resorption may make trouble for durable outcomes; (3) some surgical methods are also correlative to specific disadvantages, such as complex steps, long treatment cycle, and pin site infection [1, 9]. These limitations have put forward a higher demand for an ideal bone substitute and therapeutic technique to improve the present situation, which should be both technologically tractable and economically acceptable.

With rapid reform and innovation of biomedical metallic materials and additive manufacturing (commonly known as 3D printing), fabricating custom scaffolds to replace traditional bone substitutes for repairing critical defects has become unprecedentedly accessible and affordable [10, 11]. Synthetic metallic implants are appropriate candidates for maintaining initial and long-term mechanical stability since their stiffness and structure can be adjusted on demands [12, 13]. Relying on the 3D printing, complex geometric configuration and micro architecture can be created by altering the computer-aided design (CAD) via a layer-by-layer pattern [13–15]. Among numerous biomedical metallic materials, titanium (Ti) and its alloys have exhibited more preponderances due to excellent biocompatibility, acceptable ductility and plasticity, satisfactory mechanical strength, and superior corrosion resistance [12, 13]. Tetsworth et al. [9] once attempted to apply patient-specific 3D Ti-truss with bone graft packed to treat femoral defects and found some newly formed bone bridging around the implant. Pobloth et al. [16] also applied 3D Ti-mesh to repair bone defects which were caused by tumorous destruction and nonunion after trauma. Nevertheless, these existing scaffolds in clinical use were often equipped with the architecture of polygonal latticed truss or cage rather than dense porous structure, and to some extent, new bone regeneration and fusion are bound up with the ABG packed inside. Under this circumstance, the pattern of defect repair and new bone regeneration after 3D printed porous Ti6Al4V scaffold implantation without bone grafting still requires further research.

In our previous study, we have reported that the positive clinical effects of the 3D printed porous scaffold implantation in treating large metaphyseal femoral bone defects [17], including new bone regeneration and functional restoration. In this study, we further explored the feasibility and effectiveness of 3D printed porous Ti6Al4V scaffold without loading bone grafts in treating diaphyseal defects of lower limbs. Actually, because of lacking cancellous bone distribution, the new bone regeneration become more difficult in diaphyseal region theoretically. With more than 1-year clinical follow-up, we paid close attention to some noteworthy issues: (1) feasibility and effectiveness of our therapeutic method in clinical application, especially for patients suffering from osteomyelitis and nonunion with poor osseous and vascular structure; (2) patterns and characteristics of new bone regeneration in critical diaphyseal bone defects of lower limbs treated by our reconstruction method; (3) stability mechanism of weight-bearing limbs with critical defects, whether early functional training and weight-bearing could be achieved, as well as realizability conditions; (4) long-term outcomes and related complications. Our collective experience of this study can accumulate valuable theoretical and practical data, providing novel references for surgical regimen.

## Materials and methods

### Patients selection and therapeutic process

From December 2017, we started this prospective study, which mainly aimed to explore the clinical feasibility and effectiveness of patient-specific porous Ti6Al4V scaffold implantation combined with Masquelet technique in treating diaphyseal bone defects of lower limbs. The clinical study was approved by the Ethics Committee of Peking University Third Hospital (Beijing, China; Approval No. M2018174), and conducted in accordance with national guidelines. Patient inclusion criteria are as follows: (1) bone defects caused by nonunion or osteomyelitis; (2) the length of defects was more than 6 cm; (3) patients aging more than 18 years old; (4) follow-up period continued for more than 1 year. Patient exclusion criteria are as follows: (1) bone defects caused by tumor removal; (2) bone defects related to metaphyseal part; (3) allergy to prosthetic components materials; (4) patients unwilling or unable to finish the follow-up.

The included patients were preoperatively evaluated with a complete history of illness, especially current or previous infection and comorbidities. Preoperative laboratory tests included testing of C-reactive protein level and erythrocyte sedimentation rate. The radiographic examination included X-ray, computed tomography (CT), magnetic resonance imaging, and radionuclide bone imaging for deformity evaluation and preoperative planning. In addition, patients should be examined and treated for any nutritional or metabolic deficiencies, immune disease and other comorbidities impacting healing. The whole surgical process was divided into two stages:

1. The initial stage required thorough debridement, preliminary stabilization, and application of the Masquelet technique incorporation with antibiotic-loaded polymethyl-methacrylate (PMMA) cement as a temporary spacer. Infected bone and sequestrums were gradually cleared until cortical bleeding (namely paprika sign), which was regarded as an end-point of bone resection. Several targets exist in this stage: (1) removal of all untight or chronically infected fixation devices; (2) eradication of all infected or non-vital bone and soft tissue; (3) multi-spot biopsies (minimum of 3–5 specimens) for culture and sensitivity to guide antibiotic application. After the bone defect was cleared, an antibiotic-loaded PMMA spacer was configured and inserted with a coverage to bone ends to provide more sufficient induction of the membrane and further recipient chamber. The wound was closed by primary suture, vacuum sealing drainage or facial reconstructive surgery. A temporary external fixator was utilized to maintain immediate stability, which was rigid enough to facilitate early mobilization and unrestricted motion.
2. During the interval between two stages, several repeated debridement and bacterial cultures were implemented with the replacement of PMMA Spacer. According to different illnesses and physical conditions, the interval lasted for ~8–12 weeks, which could provide enough time for healing soft tissue injuries, course of chemotherapy, and induced membrane developing appropriately. The induced membrane was described as a biological entity with fibroblastic cells, containing high concentrations of growth and osteogenic factors [18], possessing an ability to promote new bone regeneration [19]. We did not implant additional ABG inside or outside the scaffold. New bone may develop from the defect ends in the interval time. During the process of spacers exchange and scaffold implantation, protection of the integrity of induced membrane was extremely vital.

Postoperatively, patients were permitted early functional mobilization according to specific conditions. We set the permission criteria including patients who were not affected by osteoporosis and who owned thick and strong diaphyseal cortical bone. This could be monitored and judged by CT examination. Those with poor newly formed bone or obvious osteoporosis were recommended to extend the non-weight-bearing period. Loading was added under professional guidance, from partial to full weight-bearing. At each time point, wound healing conditions were reviewed in terms of swelling, exudation, and infection. Besides, joint movement degree, walking manner, and activities of daily living were also analyzed and recorded. Routine X-ray and CT were undertaken to assess bone reconstruction and ingrowth, as well as associated complications.

### Design and fabrication of the scaffolds

**Fig. 1:**
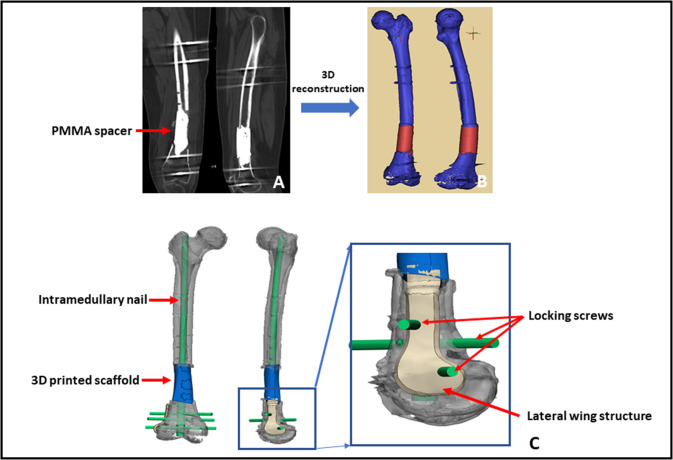
The design process of 3D porous scaffold. **A** CT scan images of the femoral shaft with PMMA spacer; **B** 3D reconstruction was performed based upon CT scan; **C** the suitable scaffold model design and its fixation by intramedullary nail was realized by means of medical-industrial interactive platform; **D** the distal side of the scaffold was fixed by locking screws and lateral wing structure

Before discharge, patients need to perform high-precision CT and X-ray to prepare for the following scaffold design. We designed the patient-specific scaffold with the aid of special anatomic landmarks around the defect and natural skeletal structures from the contralateral side showed by preoperative bilateral CT and X-ray images. Standard Ti6Al4V powers were selected as fundamental components of the current scaffold because they have been applied as favorable biomaterials in orthopedic realm for long-time and suggested to enhance osteoblast differentiation and accelerate osseointegration by a number of scholars [20, 21]. Besides, the non-absorbable property of Ti could provide a durable biomechanical environment, avoiding desynchrony between material degradation and new bone growth. The scaffolds were fabricated via selective laser melting (Arcam EBM, Gothenburg, Sweden) directed by the CAD data. We successfully obtained the 3D dense porous scaffolds with these characteristics, including elasticity modulus of 1200 ± 48 MPa, pore size of 625 ± 70 μm, high interconnectivity, and porosity of 68%. The elastic modulus of normal bone is 0.5–20 GPa [22], and our current scaffold was similar to trabecular bone, while it is significantly lower than cortical bone, which is highly advantageous to eliminate the stress shielding [23]. Regarding pore size and porosity, Taniguchi et al. [24] reported that the porous Ti6Al4V implant with a designed porosity of 65% and a pore size of 600 μm had more comparable mechanical strength with the bone, as well as further bone ingrowth than those with a pore size of 300 and 900 μm. Thus, the architecture of our present scaffold facilitated superior biomechanical support and bone regeneration. The central part of the scaffold was chiseled as a cylindrical hole to accommodate subsequent intramedullary (IM) nail insertion. After fabrication, all scaffolds were cleaned in an ultrasonic bath, so as to remove all loose titanium particles and impurities. Finally, they were sterilized and packed for surgery. The detailed design process of 3D personalized porous scaffold can be referred to Fig. 1.

## Results

In total, ten patients (11 parts of bone defects) whose follow-up period exceeded 1 year were enrolled in this study. Their clinical and follow-up characteristics are listed in Table 1. The case series included three females and seven males, and their median age was 50.7 years old. Causes of defects included osteomyelitis (7 parts) and aseptic nonunion (4 parts), and infected pathogens involved Morganella morganii mixed with Staphylococcus haemolyticus (1 case), Staphylococcus aureus (1 case), Staphylococcus haemolyticus (1 case), Pseudomonas aeruginosa (1 case) and Enterobacter cloacae (3 case). The location of defects included five parts of femur and six parts of tibia, and their average defect distance was 12.2 cm. Postoperatively, there was no significant difference between the full length of both legs measured by X-ray, and the mean difference was 0.64 (0.2–1.2) cm. In terms of radiographic follow-up, conventional X-ray displayed that new callus increasingly grew from both proximal and distal ends to the middle along the surface of scaffold. Moreover, new bone was remodeled with different degrees of hypertrophy, and new callus could develop and proliferate at both ends of the defects, forming a stable “pedestal-like” structure. Meanwhile, the ends of host bone developed some sclerosis and absorption. Additionally, nine patients were permitted to exercise postoperative weight-bearing within 2 weeks (from 5 to 14 days), while the other patient obtained permission with a prolong interval of 25 days because of her osteoporosis. During the postoperative follow-up, one patient was diagnosed with tibial varus deformity accompanied by scaffold loosening, he underwent a revision surgery and achieved correction of tibial force line. In the last follow-up, apart from one patient who walked with a stick, all cases could obtain limb salvage and independent walking. The result of wound healing was satisfactory, and recurrence of infection did not appear as well. No another migration and breakage of scaffolds and internal fixation were observed.

**Table 1:** Clinical characteristics of typical patients from our hospital

	Age (years)/ Gender	Causes (pathogen)	Location (distance)	Postoperative full lower limb length (cm)			Postoperative interval to weight-bearing	Follow-up (months)/Outcomes
				L	R	Difference		
1	27/M	Osteomyelitis (MM, SH)	R-Femur (12.9 cm)	82.1	81.9	0.2	5 days	35/new bone formation and scaffold stability
2	32/M	Osteomyelitis (EC)	L-Tibia (12.4 cm)	77.6	78.4	0.8	10 days	14/new bone formation and scaffold stability
3	43/M	Osteomyelitis (SA)	L-Tibia (6.3 cm)	88.3	87.1	1.2	5 days	35/new bone formation and scaffold stability
4	61/M	Osteomyelitis (EC)	R-Femur (14.0 cm)	85.6	84.9	0.7	10 days	23/new bone formation and scaffold stability
5	65/M	Osteomyelitis (EC)	R-Tibia (10.6 cm)	81.9	81.7	0.2	13 days	24/new bone formation and scaffold stability
6	67/F	Osteomyelitis (SH)	R-Femur (13.3 cm)	79.2	78.9	0.3	14 days	13/new bone formation and scaffold stability
7	37/M	Osteomyelitis (PA)	L-Tibia (10.8 cm)	80.5	81.1	0.6	12 days	16/new bone formation but tibial varus deformity
8	43/F	Aseptic nonunion	R-Femur (13.4 cm)	76.2	77.1	0.9	10 days	27/new bone formation and scaffold stability
		Aseptic nonunion	R-Tibia (8.5 cm)					
9	69/F	Aseptic nonunion	L-Femur (9.5 cm)	71.7	71.1	0.6	25 days	30/new bone formation and scaffold stability
10	63/M	Aseptic nonunion	R-Tibia (22.9 cm)	80.5	79.6	0.9	14 days	13/new bone formation and scaffold stability

*M* male, *F* female, *L* left, *R* right, *MM* Morganella morganii, *SH* Staphylococcus haemolyticus, *SA* Staphylococcus aureus, *EC* Enterobacter cloacae, *PA* Pseudomonas aeruginosa

With regards to the postoperative complications, one patient respectively experienced locking screw loosening and breakage, and one patient experienced angulation deformity.

**Fig. 2:**
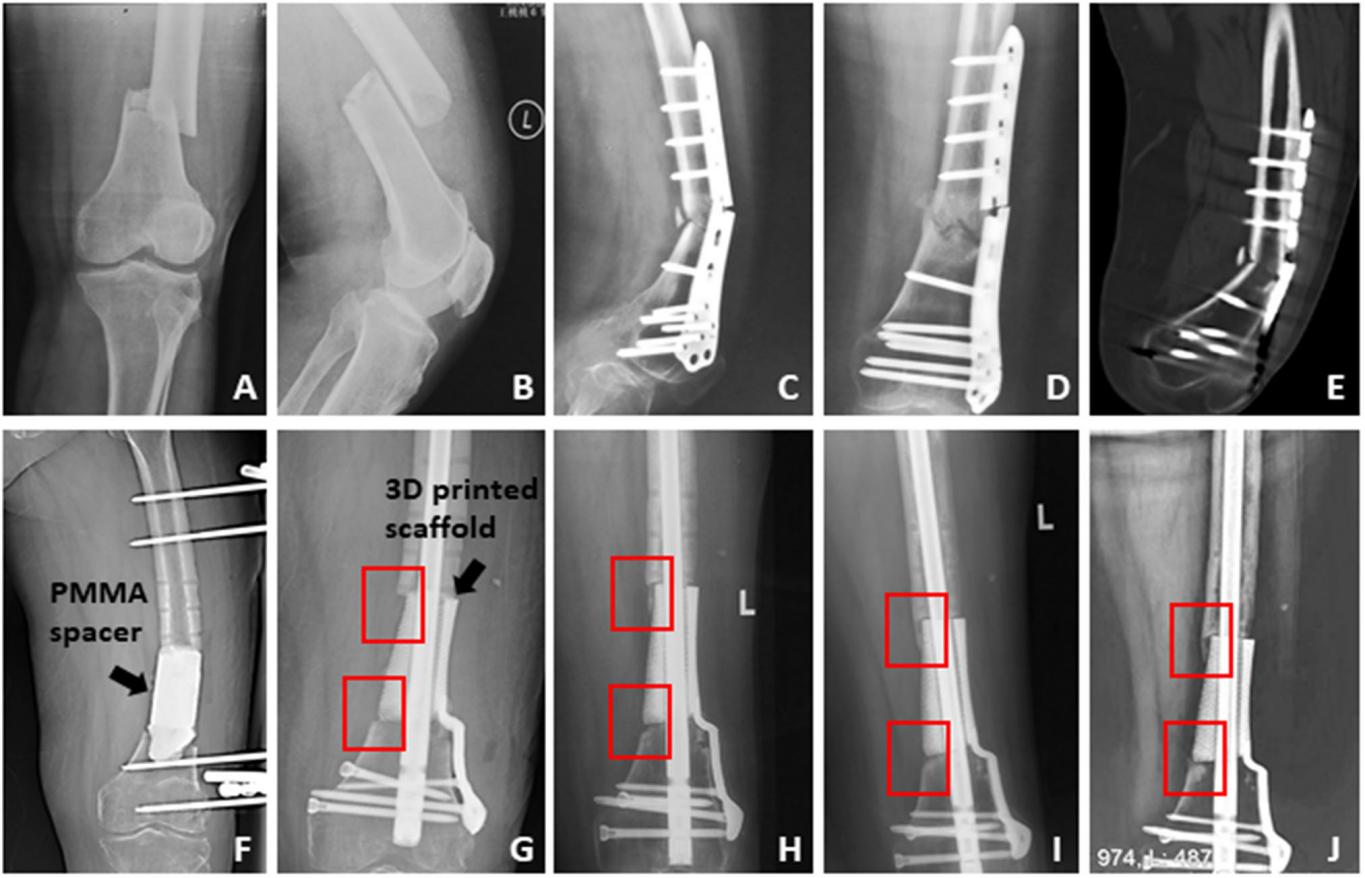
The therapeutic process of the first typical case. **A**, **B** The initial X-ray displayed the patient suffered the distal femoral displaced fracture; **C**–**E** The patient underwent the surgical treatment, and at 13 months after the initial surgery, X-ray and CT scan showed fracture nonunion and ends sclerosis, meanwhile, the plate had broken; **F** we cleared the sequestrums and filled a PMMA spacer into the femoral defect with 9.5 cm length, and stabilized the femur by external fixator; **G**–**J** after a 10-week interval, the bone defect was repaired by a customized 3D printed scaffold, and the postoperative regular X-rays showed the scaffold kept stable and new bone grew gradually on the surface of scaffold from both ends of the defect (as displayed in the red boxes)

**Fig. 3:**
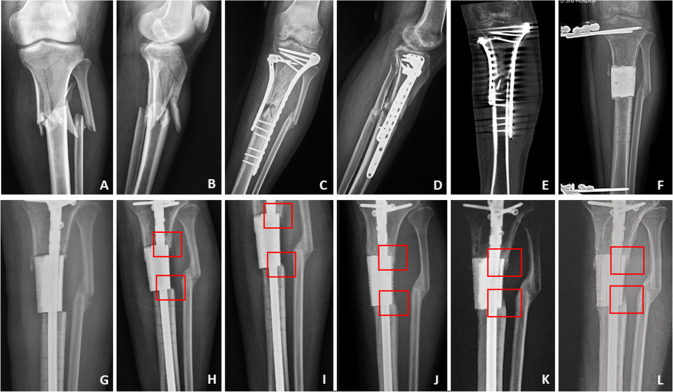
The therapeutic process of the second typical case. **A**, **B** The initial X-ray displayed the patient suffered the tibial comminuted fracture; **C**–**E** the patient underwent the surgical treatment, and at 10 months after the initial surgery, X-ray and CT scan showed fracture nonunion and sequestrums; **F** we cleared the sequestrums and filled a PMMA spacer into the femoral defect with 6.3 cm length, and stabilized the femur by external fixator; **G**–**L** after an 8-week interval, the bone defect was repaired by a customized 3D printed scaffold, and the postoperative regular X-rays also showed the scaffold kept stable and new bone grew gradually on the surface of scaffold from both ends of the defect (as displayed in the red boxes)

Two typical cases were displayed:

1. The first patient was a female with the age of 69 years old (Case 9 in Table 1). She suffered from open femoral fracture (Fig. 2A, B) after a traffic accident and underwent surgery 13 months before her admission to our hospital. Unfortunately, she encountered aseptic nonunion and plate breakage (Fig. 2C–E). As shown by Fig. 2C–E, osteosclerosis and hyperplasia occurred at the fracture ends. After thorough debridement, osteotomy and spacer replacement (Fig. 2F), a critical femoral defect of 9.5 cm was repaired by a 3D printed porous scaffold without additional bone grafting. We could observe newly formed bone formation from both ends to middle and robust scaffold location during the follow-up period of 30 months (Fig. 2G–J).

(2) The second patient was a male who aged 43 years old (Case 3 in Table 1), and he suffered from infected nonunion of the tibia after a fixation surgery for comminuted fracture (Fig. 3A, B). After the routine preparation procedure, his tibial bone defect (6.3 cm) was also repaired by a 3D patient-specific scaffold without additional bone grafting. Regular radiographies showed newly gradually formed bone and stable scaffold location (Fig. 2G–L). At 35 months after surgery, his weight-loading ability and knee motion (bent or straight) of both legs were satisfactory (Fig. 3).

## Discussion

**Fig. 4:**
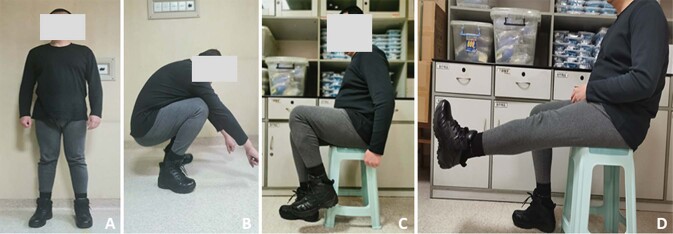
The lower limb function of the second typical case at the last follow-up. As displayed, his loading and motion function of both legs were satisfactory. **A** Stand, **B** Crouch, **C** Bend leg, and **D** Straight leg

In general, bone is a dynamic tissue that is constantly being remodeled, which is generally stimulated and instructed by well-balanced biological and microenvironmental elements. However, when a defect reaches a non-healable size, external intervention may become necessary to supply self-healing. Despite recent advances made in biomaterial and surgical methods, a great number of patients had undergone amputations each year because traditional methods contain a number of limitations and complications. Therefore, repair of critical bone defects of lower limbs still requires an effectively surgical and practical method. In the present study, we accomplished to combine Masquelet technique and 3D printed porous Ti6Al4V scaffold implantation without additional bone grafting to treat critical diaphyseal bone defects. Although a number of scholars have successfully implemented the repair of complex limb defects by 3D prosthesis implantation [9, 16, 25–27], our treatment approach exhibited a clear difference from those previous methods. Several scholars added ABG inside scaffolds to promote new bone formation, while our treatment procedure abolished the need for additional ABG, which could alleviate complications caused by bone grafting. Taken together, our previous [17] and present studies synthetically revealed the effectiveness of 3D printed scaffold implantation in treating critical bone defects of lower weight-bearing limbs (Fig. 4).

According to the above-mentioned clinical results, without implantation of additional bone grafting, new bone could gradually regenerate, and some obvious characteristics could be observed. On the one hand, originating from both ends, new bone callus could grow on the surface of porous scaffold at the junctional zone, connecting both scaffold and adjacent host bone. On the other hand, we could also observe dense bone hypertrophy and callus proliferation at both ends of the defect. During the early stage of bone regeneration, stress from local micromotion at the junctional zone caused reactive bone proliferation. In the following stage, with the gradual remodeling of trabeculae in cancellous bone along the direction of force and reconstruction of new bone, the interface tended toward biological bone fusion. The newly formed pedestal-like structure could provide stable mechanical support for scaffold stability and the following callus crawling. Furthermore, blood supply and soft tissue coverage exerted a crucial influence on the newly formed bone, and further newly formed callus could be observed at the region with relatively abundant blood supply, including posterior side of the scaffold and side close to metaphysis, in addition to femoral region compared with tibia.

In the current clinical study, all patients were allowed to implement early weight-bearing once conditions permit. Apart from patients’ intrinsic physical conditions with adequate bone mineral density, some efforts were also made to guarantee the safety and success during recovery process. We designed a rough and angular surface of both ends of the 3D scaffolds to strengthen friction and roughness, which could act as an interfacial lock once the scaffold contacts the host bone during early weight-bearing. In order to achieve long-term stability of implants and less loosening and failure, the fulfillment of stable mechanical support and osteointegration are essential. The former has been realized based on the clinical manifestations with new bone surrounding the scaffold from both ends. In the last follow-up, no severe complications (e.g., scaffold breakage or fixation failure) occurred during postoperative period up to 35 months. However, all the patients were unable to walk completely normal and suffered from different levels of claudication symptoms, which might be related to the preoperative degenerated function of stiff joints due to long period of inactivity. Moreover, a number of patients expressed that they were afraid of fixation failure caused by radical weight-bearing and abnormal walking. Hence, it is significant to encourage them for properly early rehabilitation and eliminating their postoperative psychological burdens to assist them to get back to normal activities.

In order to effectively achieve success with the aid of the present treatment method, three surgical key points need to be emphasized: (1) thorough debridement (directed by paprika sign) of sequestrum and decayed bone for nonunion and osteomyelitis is essential, and adequate blood supply from the active bleeding of the cutting edge can guarantee the subsequent new bone regeneration, while limitations created by the size and morphology of the defect become roughly neglectable as long as the customization advantages of 3D printing can be sufficiently maximized; (2) IM nail is more advocated to fix the scaffold in diaphyseal region because of its advantage related to biomechanical conduction in lower limbs, while plate may be biomechanically unfavorable in the presence of a defect due to cantilever loading and tension band principle, and then, axis deviation or breakage may occur during motion and loading [16, 28]; (3) preservation of an intact induced membrane is highly essential to promote new bone formation, because it possesses three important functions [18], including secreting regenerative factors and signals to modulate cell behavior and promote bone regeneration, providing a vascular network to supply nutrients and oxygen, and serving as a barrier to impede soft tissue invasion and new bone resorption.

The present study has a number of limitations. Firstly, the present study included a relatively limited number of patients, making a difficulty to achieve deeper research results related to new bone regeneration and clinical effects. Furthermore, it is still difficult to precisely describe whether new bone is able to grow inside the scaffolds after implantation based on the existing examinations. Further research is required to enroll more patients and acquire longer follow-up data from multi-center and comparative studies.

## Conclusion

In the current research, we successfully carried out an in vivo study of 3D printed porous Ti6Al4V scaffolds to repair critical diaphyseal bone defects via clinical practice. Without implanting additional bone grafts, new callus could regenerate with some characteristics at the region with relatively abundant blood supply, mainly including on-growth of the scaffold, dense hypertrophy, and proliferation at both ends of defects. Besides, the trabeculae of cancellous bone can thicken and remodel along the force direction at the junctional zone. These characteristics may provide guarantee reliable reference for stable mechanical environment and long-term functional rehabilitation. Therefore, the proposed innovative treatment technique is an effective option for reconstructing critical diaphyseal bone defects of lower weight-bearing limbs.

## Data Availability

The data used to support the results of this study are available from the corresponding author upon request.

## Abbreviations

3D: Three dimensional
ABG: Autogenous or allogeneic bone graft
CAD: Computer-aided design
Ti: Titanium
CT: Computed tomography
MRI: Magnetic resonance imaging
RNBI: Radionuclide bone imaging
IM: Intramedullary
